# Detonating a “time bomb”: how enteric infection stress triggers prophage induction in commensal microbiota and exacerbates disease progression

**DOI:** 10.3389/fmicb.2025.1723549

**Published:** 2026-01-06

**Authors:** Ling Yang, Yan Xu, Xinna Wang

**Affiliations:** 1 School of Public Health and Health Management, Qujing University of Medicine & Health Sciences, Qujing, China; 2 School of Pediatrics, Henan University of Chinese Medicine, Zhengzhou, China; 3 Department of Encephalopathy, Affiliated Hospital of Changchun University of Chinese Medicine, Changchun, China

**Keywords:** commensal microbiota, defensive barrier, enteric infection stress, exacerbates disease, prophage

## Abstract

The pathophysiology of enteric infections involves more than a straightforward pathogen–host interaction; a critical yet often underappreciated factor is the functional shift of the commensal microbiota in response to stress. This review constructs and substantiates a “Prophage-Mediated Implosion Hypothesis” to provide an integrative framework for explaining the complexities of gut infections. This hypothesis posits that during enteric infection, the stress microenvironment—generated by the pathogen, host immune responses, and clinical interventions—transforms the commensal microbiota from a “defensive barrier” into a “destructive endogenous amplifier” via prophage activation. This article systematically elucidates the three core stages of this “implosion” process: (1) how key signaling networks, represented by endogenous DNA damage (e.g., reactive oxygen species [ROS]) and exogenous agents (e.g., antibiotics), trigger the SOS response and activate prophages; (2) how prophage activation disrupts colonization resistance (via commensal lysis), mediates the horizontal transfer of virulence and resistance genes, and exacerbates inflammation (via PAMPs and the TLR9 pathway), thereby creating a destructive cascade; and (3) how this hypothesis offers novel mechanistic explanations for clinical challenges such as antibiotic-associated complications and the heterogeneity in infection severity. Finally, building on this framework, the review discusses emerging intervention strategies—such as antibiotics that spare or support bacteriophage activity and therapies targeting the SOS response to attenuate bacterial virulence. This work aims to shift the understanding of enteric infections from a traditional “external invasion” model to an integrated “combined internal and external assault” model.

## 1 Introduction

Enteric infectious diseases represent a major global public health challenge, with treatment heavily reliant on antibiotics (Talaat et al., 2019). However, the widespread use of antibiotics accelerates antimicrobial resistance, frequently induces severe gut dysbiosis, and can paradoxically worsen the disease. This reveals a critical knowledge gap in our understanding of the pathophysiology of enteric infections. Current research paradigms primarily focus on two perspectives: one centers on the “pathogen-host” binary conflict, exploring how pathogens evade host immunity; the other, from a microbial ecology perspective, the focus lies on the competition for resources and the role of colonization resistance in the interaction between pathogens and the commensal microbiota. While vital, these perspectives generally regard the commensal microbiota as a passive “defensive barrier” or “victim,” overlooking its potential to transition to an active, destructive role under specific stress conditions. Dispersed evidence from virology, microbiology, and immunology collectively points to an underappreciated key mechanism: the activation of prophages integrated within the genomes of commensal bacteria.

**Figure 1:**
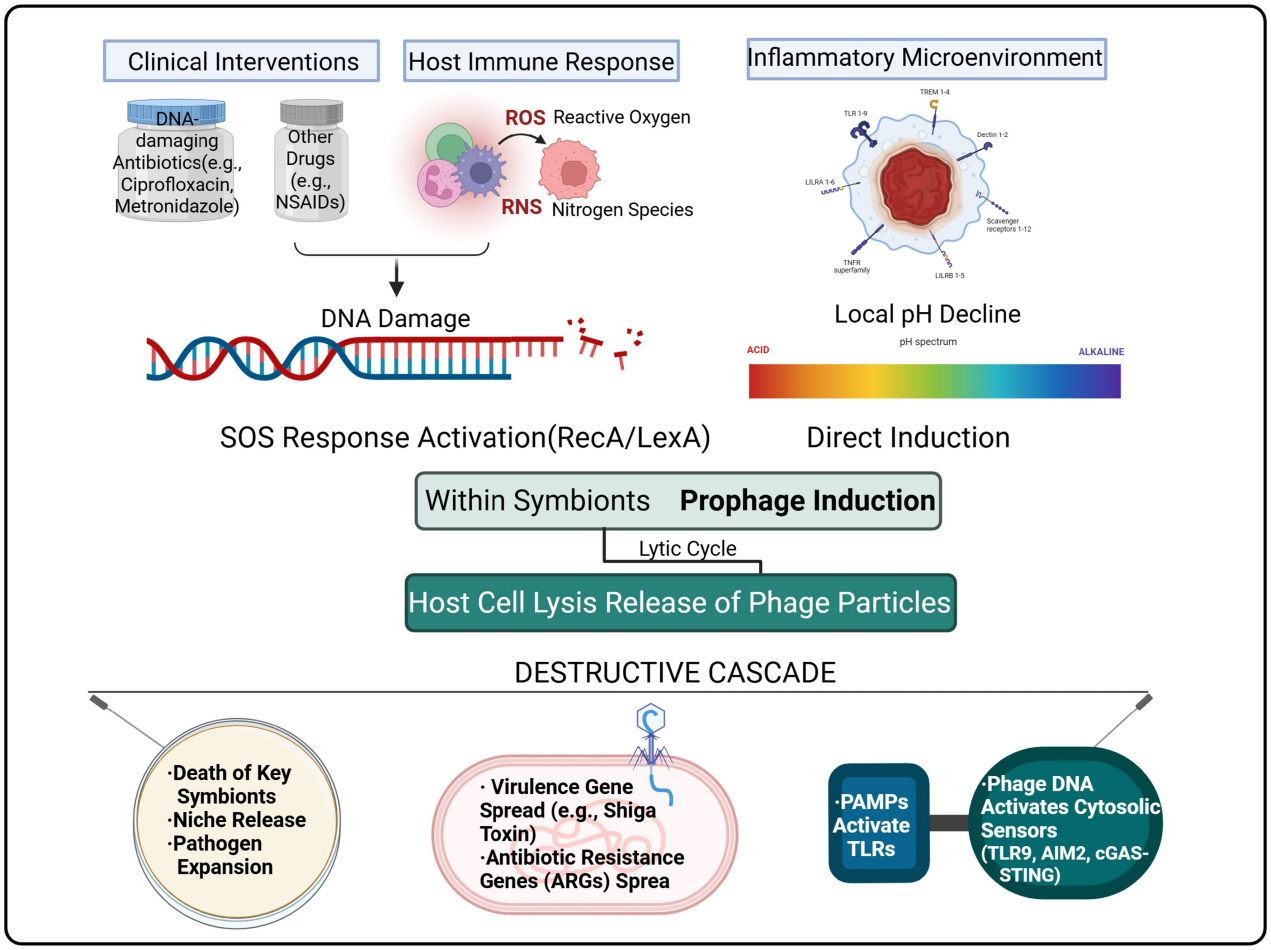
Triggering signals: a network of inducers, including clinical interventions (e.g., DNA-damaging antibiotics), host immune responses [e.g., reactive oxygen species (ROS)], and the inflammatory microenvironment (e.g., low pH), converges primarily on the activation of the SOS response pathway, triggering prophage lysis. Central hub: prophages integrated within commensal bacterial genomes are activated, leading to mass lysis of host bacteria and the release of numerous mature phage particles. Cascading destructive effects: this “implosion” process exacerbates the disease through three main pathways: (1) disruption of colonization resistance by lysing key commensal bacteria, creating ecological niches for opportunistic pathogen expansion; (2) acceleration of horizontal transfer of virulence and resistance genes within the microbiota through mechanisms like generalized transduction; and (3) exacerbation of the host inflammatory response through the concentrated release of pathogen-associated molecular patterns (PAMPs) (e.g., lipopolysaccharide) and phage DNA (a TLR9 agonist), leading to over-activation of the innate immune system.

Building on this, this review aims to construct and systematically elaborate a unified theoretical framework—the “Prophage-Mediated Implosion Hypothesis.” The central tenet of this hypothesis is that during an enteric infection, the complex stress environment—composed of the pathogen, host immune response, and clinical treatments—transforms the commensal microbiota, originally a defensive barrier, into a “destructive endogenous amplifier” mediated by prophage activation. This “implosion” process contributes to disease exacerbation through multiple pathways: lysis of key commensal bacteria, accelerated horizontal transfer of virulence and resistance genes, and intensification of the inflammatory response. Therefore, this review integrates isolated evidence from across disciplines to provide a novel explanatory model—based on the microbiome’s internal dynamics—for complex clinical phenomena, such as antibiotic treatment failure and heterogeneity in infection severity. The specific objectives of this article are to systematically argue for the three core components of the “implosion hypothesis”: (1) to analyze the key signaling networks that trigger prophage activation; (2) to delineate the cascading destructive effects following prophage activation; and (3) to explore the profound implications of this mechanism for clinical practice. By constructing this framework, we aim to advance the understanding of enteric infection pathophysiology. This provides a theoretical foundation for developing safer, more precise, next-generation intervention strategies that target microbiome stability (see Figure 1).

## 2 The key signaling network triggering prophage “implosion”

**Figure 2:**
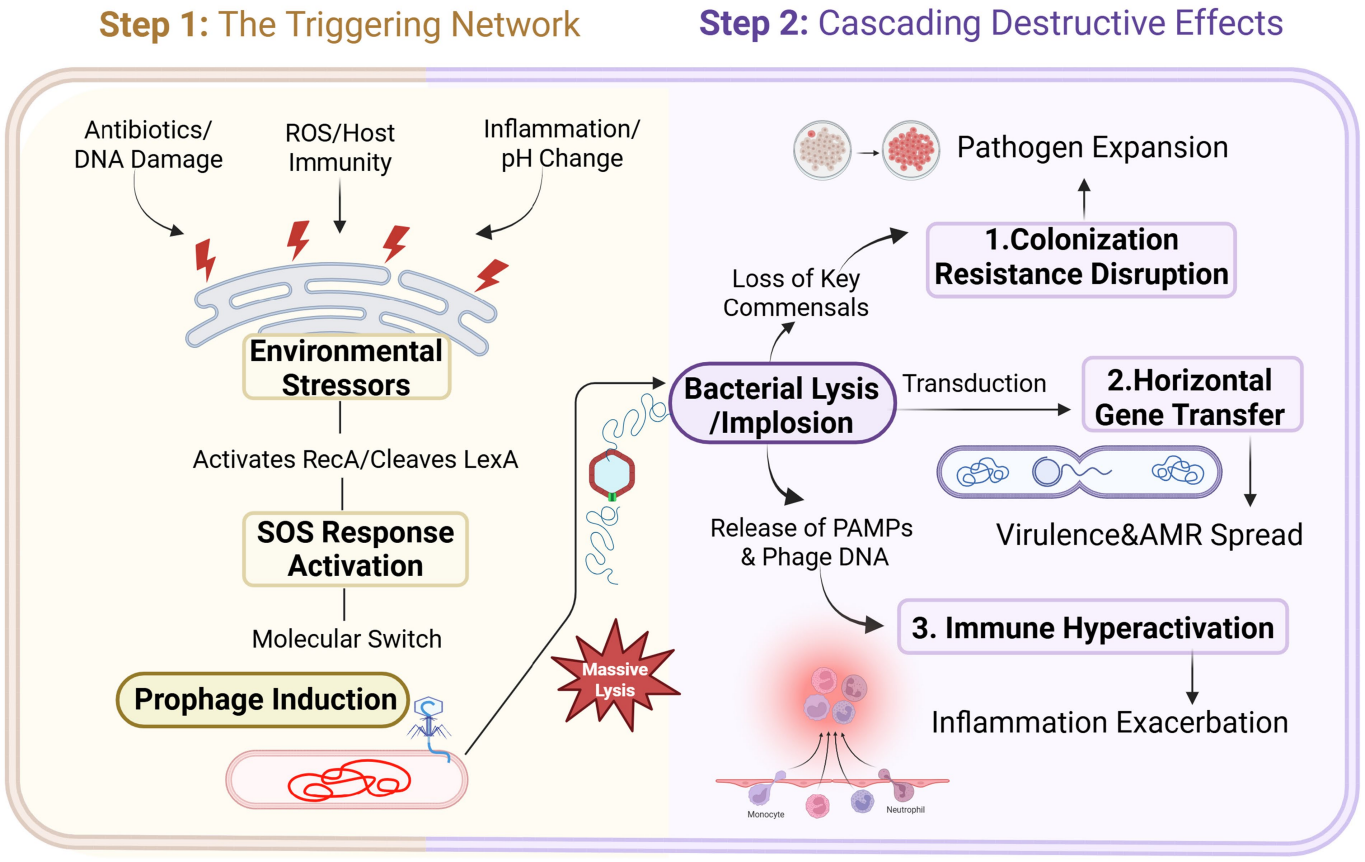
Schematic representation of the “Prophage-Mediated Implosion” hypothesis. The diagram illustrates the transition from environmental triggers to disease exacerbation. (Top) Environmental stressors such as antibiotics and ROS activate the bacterial SOS response, serving as the molecular switch for prophage induction. (Bottom) The resulting mass lysis of commensal bacteria triggers three cascading destructive effects: disruption of colonization resistance, acceleration of horizontal gene transfer (HGT), and exacerbation of host inflammation via PAMPs and phage DNA release.

Prophage activation is not a random event but a precise response to specific environmental stress signals. In the complex microenvironment of an enteric infection, signals from the host, pathogen, and clinical interventions intertwine to form a potent induction network, collectively pulling the “trigger” for prophage lysis (Figure 2).

### 2.1 The SOS response: the core molecular switch for prophage activation

The SOS response, a primary repair mechanism for bacteria facing DNA damage, is also the most central and well-understood molecular switch for prophage activation. It hinges on two core proteins: the sensor RecA and the repressor LexA. Under normal physiological conditions, dimeric LexA binds to the operator regions of multiple SOS response genes, including prophage lytic genes, strongly inhibiting their transcription. However, when DNA damage (e.g., double-strand breaks) occurs, the signal is sensed by RecA, which then polymerizes on single-stranded DNA (ssDNA) fragments to form a nucleoprotein filament (RecA) with coprotease activity. This activated RecA catalyzes the autocleavage and inactivation of LexA and various phage-encoded repressors (e.g., the CI protein). The degradation of these repressors lifts the inhibition on lytic genes, thereby initiating the process of prophage excision, replication, and eventual host lysis (Little, 1984). Consequently, any factor that can directly or indirectly cause DNA damage in commensal bacteria can serve as an upstream signal for triggering prophage activation.

### 2.2 Host immunity and the inflammatory microenvironment: endogenous triggers of the SOS response

In combating pathogens, the host immune system inadvertently becomes a powerful driver of prophage activation in the commensal microbiota. For instance, neutrophils and macrophages, as the first line of immune defense, produce large amounts of reactive oxygen species (ROS) and reactive nitrogen species (RNS), such as hydrogen peroxide and nitric oxide (NO) (Fang, 2004). These highly oxidative molecules can cause DNA strand breaks or base modifications, acting as potent endogenous DNA-damaging agents that activate the SOS response (Kimmitt et al., 2000). Furthermore, complex microenvironmental changes induced by intestinal inflammation, such as a significant drop in local pH, can also act as independent signals to induce prophage activation. This effect may be related to the acidic environment’s impact on the conformation and stability of phage repressor proteins (Shuwen and Kefeng, 2022).

### 2.3 Clinical interventions: DNA-damaging drugs as potent inducers

Iatrogenic factors, particularly clinical interventions, are another major driver of prophage activation. Among these, the use of antibiotics constitutes the most direct and potent source of induction. The main antibiotics known to induce prophages are summarized in Table 1.

**Table 1:** Examples of antibiotics and other drugs that induce prophage activation.

Antibiotic/drug class	Example	Mechanism of induction	Key finding/references
(Lab standard)	Mitomycin C	Forms DNA cross-links, induces SOS response.	“Gold standard” for verifying prophage activity (Gong et al., 2025).
Fluoroquinolones	Ciprofloxacin	Inhibits DNA gyrase, causes double-strand breaks, induces SOS response.	Induces Shiga toxin-encoding phages, increases mouse mortality (Zhang et al., 2000).
Nitroimidazoles	Metronidazole	Generates free radicals, directly damages DNA structure.	Efficiently triggers the SOS response (Muller et al., 2011).
Folate synthesis inhibitors	Trimethoprim	Inhibits folate synthesis, activates SOS response.	Can promote horizontal gene transfer (Revitt-Mills et al., 2022; Mohanraj and Mandal, 2022).
Macrolides	Azithromycin	Affects protein synthesis, activates SOS response.	Can induce SOS and promote HGT (Mohanraj and Mandal, 2022).
Non-antibiotic drugs	NSAIDs	(Mechanism varies)	Can induce prophages from human gut isolates (Sutcliffe et al., 2021).

Mitomycin C, a classic inducer in laboratory studies, induces the SOS response by forming cross-links between DNA strands; its powerful inducing effect has made it the “gold standard” for verifying prophage activity (Gong et al., 2025). In clinical practice, many widely used antibiotics are recognized as strong inducers, especially those that inhibit DNA replication or repair. For example, fluoroquinolones (e.g., ciprofloxacin) inhibit DNA gyrase, leading to stalled replication forks and double-strand breaks, while metronidazole generates DNA-damaging free radicals upon reduction. Both can efficiently trigger the SOS response (Muller et al., 2011). More concerning is that this inducing effect is not unique to a few drug classes. Studies have shown that other classes of antibiotics, such as trimethoprim, which inhibits folate synthesis, and macrolides (e.g., azithromycin), which affect protein synthesis, can also activate the SOS response and promote horizontal gene transfer, greatly expanding the range of potentially risky drugs (Revitt-Mills et al., 2022; Mohanraj and Mandal, 2022). This effect can be triggered even at sub-minimum inhibitory concentrations (sub-MIC) of antibiotics. For instance, sub-MIC levels of ciprofloxacin induce prophages in the environmental bacterium *Geobacter* (Fang et al., 2023). A landmark animal experiment clearly demonstrated that treating mice infected with *Escherichia coli* carrying a Shiga toxin-encoding prophage with ciprofloxacin led to a sharp increase in toxin production and a significant rise in mouse mortality (Zhang et al., 2000). This finding is supported by clinical retrospective studies, which have shown a significant association between the use of certain antibiotics in patients with enterohemorrhagic *E. coli* (EHEC) infections and an increased risk of the fatal complication, hemolytic uremic syndrome (HUS) (Huelsenbeck et al., 2000). Furthermore, the induction risk extends far beyond antibiotics and chemotherapy drugs. One study systematically tested a variety of common non-antibiotic drugs and found that several, including non-steroidal anti-inflammatory drugs (NSAIDs), could induce prophages from human gut isolates at gut-relevant concentrations, revealing a severely underestimated source of microbiome perturbation driven by everyday medications (Sutcliffe et al., 2021).

## 3 The cascading destructive effects mediated by prophage activation

Once activated, the synchronous lysis of numerous prophages triggers a chain reaction, akin to detonating “cluster bombs” within the microbial ecosystem. These destructive effects impact the structure and function of the microbiota and the host immune system, forming a vicious cycle that exacerbates the disease.

### 3.1 Disruption of colonization resistance and niche provision for pathogens via lysis of key commensals

The dominant commensal bacteria in the gut, particularly members of the genera *Bacteroides* and *Clostridium*, are the mainstays of “colonization resistance,” which maintains microbial stability and resists invasion by external pathogens (Buffie and Pamer, 2013). However, these key commensals are heavily populated with prophages. Metagenomic analyses have revealed that their genomes universally carry a high number and diversity of prophages (Wickman, 1986).

For example, a systematic study of the genus *Bacteroides* found not only that prophages are prevalent but also that they carry numerous genes related to antibiotic resistance and virulence and exhibit significant temporal stability (Stopnisek et al., 2025). These integrated phages are not merely silent genetic elements but are key drivers shaping their host’s genome and epigenome; for instance, a large number of genes related to DNA methylation are located in the prophage regions of *Bacteroides fragilis* (Tisza et al., 2023). Under infection-related stress, the concentrated induction of prophages leads to the mass lytic death of these key strains. This process directly reduces the biomass of beneficial bacteria and, more importantly, rapidly vacates the physical space and nutritional niches they occupied. This “niche vacuum” creates ideal conditions for the expansion of opportunistic pathogens, such as *Clostridioides difficile* (Smits et al., 2016). Furthermore, the loss of key commensal functions further weakens host defenses. A typical example is the lytic death of many butyrate-producing commensals, which leads to a significant decrease in butyrate concentration in the gut. Butyrate is the primary energy source for colonocytes, and its reduction directly impairs the integrity and function of the intestinal barrier, making the host more susceptible to pathogens (Parada et al., 2019).

### 3.2 Acceleration of horizontal transfer of virulence and resistance genes via transduction

While functioning as intrinsic antimicrobial agents, activated prophages also act as highly efficient vectors for Horizontal Gene Transfer (HGT). Many of the most lethal bacterial toxins (e.g., diphtheria, botulinum, cholera, and Shiga toxins) are encoded not on the bacterial chromosome but as “cargo” on prophage genomes—the most dangerous aspect of “lysogenic conversion (Matsunaga and Jaehning, 2004).

Furthermore, the prophage lytic cycle is a key mechanism driving the spread of antibiotic resistance genes (ARGs) in the microbiota. During lysis, errors in the phage packaging system can lead to the mispackaging of host chromosomal fragments (which may contain ARGs) into new phage particles, a process known as “generalized transduction.” These phage particles, now carrying resistance genes, can then infect other susceptible bacteria, thereby spreading the resistance trait and greatly accelerating the evolution of resistance throughout the entire microbial community (Enault et al., 2017).

### 3.3 Exacerbation of host inflammatory response via release of PAMPs and activation of cytosolic DNA sensors

Synchronous prophage activation amplifies the host inflammatory response through multiple pathways. First, the simultaneous lysis of numerous bacteria leads to a massive release of bacterial structural components (PAMPs), such as lipopolysaccharide and peptidoglycan. These PAMPs are recognized by Toll-like receptors (TLRs) on host immune cells, triggering a strong inflammatory response and potentially a cytokine storm (Mogensen, 2009). However, a deeper level of inflammatory amplification stems from the direct recognition of phage DNA, which acts as a key danger signal activating multiple intracellular DNA sensors. For instance, phage DNA, rich in unmethylated CpG motifs, is a potent agonist for Toll-like receptor 9 (TLR9). When endocytosed by antigen-presenting cells (e.g., dendritic cells), phage DNA is recognized by endosomal TLR9, which activates the MyD88-dependent pathway and leads to massive production of Type I Interferons (IFNs) (Bollyky and Secor, 2019).

On the other hand, when phages replicate within the cytoplasm of commensal bacteria and cause their lysis, large amounts of phage DNA are released directly into an environment accessible to host cells, thereby activating cytosolic DNA sensing pathways. Among these, the AIM2 (Absent in Melanoma 2) inflammasome is a key sensor. Research has clearly demonstrated that DNA released from bacteria lysing within the macrophage cytosol—for various reasons including phage lysis or antibiotic action—can be directly recognized by AIM2. This leads to the assembly of the inflammasome, activation of Caspase-1, and induction of pyroptosis, a highly pro-inflammatory form of cell death (Sauer et al., 2010). Additionally, the cGAS-STING pathway, central to antiviral innate immunity, has ancient origins in bacteria (the CBASS system) precisely to defend against phage invasion (Morehouse et al., 2020). In mammalian cells, there is direct evidence that phage DNA can be recognized by cGAS, although the activation and regulation of its downstream signaling exhibit complexity (Podlacha et al., 2024).

It is noteworthy that the interaction between phages and host immunity does not always lead to inflammation. Some studies indicate that highly purified phage particles, upon endocytosis, may not trigger the cGAS-STING or TLR9 pathways and may instead modulate cellular metabolism through other mechanisms (Bichet et al., 2023).

This suggests that the immunogenicity of phage DNA may be highly dependent on its mode of release (intracellular lysis vs. intact particle endocytosis), modification state, and host cell type, constituting an important direction for future research.

## 4 Discussion

This review constructs and substantiates the “Prophage-Mediated Implosion Hypothesis,” providing an integrative framework for understanding enteric infection pathophysiology beyond the traditional “exogenous invasion” model. We have reviewed the evidence supporting this hypothesis: the environment of DNA damage, oxidative stress, and inflammation during enteric infections constitutes a “perfect storm” for prophage activation. In turn, prophage activation transforms the commensal microbiota from “defender” to “disease amplifier” via commensal lysis, virulence gene dissemination, and inflammation exacerbation. This hypothesis not only offers a new perspective for explaining several long-standing clinical puzzles but also points to future directions for research and intervention strategies.

### 4.1 Explanatory power of the “implosion hypothesis” for clinical phenomena

The core value of the “implosion hypothesis” is its ability to explain clinical phenomena that traditional models cannot fully cover. A classic example is the significant association between using strong SOS inducers (e.g., ciprofloxacin) during EHEC infections and an increased risk of HUS (Huelsenbeck et al., 2000).

The traditional view struggles to explain why bactericidal action would worsen the condition. The “implosion hypothesis” provides a clear mechanism: while killing some pathogens, the antibiotic simultaneously induces a massive activation of Shiga toxin-encoding prophages, leading to an implosive increase in toxin production and thus drastically aggravating the damage to the host (Zhang et al., 2000). Similarly, the hypothesis helps to understand the heterogeneity of *C. difficile* infection. The pathogenicity of *C. difficile* depends not only on its own virulence but is also strongly influenced by the background microbiota. A plausible explanation is that in some patients, specific stress environments (such as prior antibiotic exposure) not only eliminate competitors of *C. difficile* but may also trigger an “implosion” of prophages in other commensal bacteria, further compromising the intestinal barrier and immune homeostasis, thereby creating a permissive environment for *C. difficile* to flourish. Furthermore, the explanatory power of this hypothesis may extend to non-infectious diseases. For example, in studies of Parkinson’s disease, a significant decrease in the abundance of *Prevotella* in the gut microbiota of patients has been observed (Bedarf et al., 2017). Although the mechanism is complex, the “implosion hypothesis” offers a perspective worth exploring: could chronic, low-grade intestinal inflammation or other endogenous stressors lead to the chronic depletion of specific bacteria like *Prevotella* through the continuous induction of their prophages, thereby participating in the pathogenesis of the disease?

### 4.2 New clinical intervention strategies based on the “implosion hypothesis”

Understanding the prophage “implosion” mechanism offers two new clinical intervention approaches beyond traditional antibiotics. The first is a “defensive strategy” aimed at “avoiding detonation”: optimizing existing therapies. This requires selecting antibiotics based not only on their antimicrobial spectrum but also on their potential to induce commensal prophages. Therefore, the development of antibiotics that preserve or support bacteriophage activity represents a critical future direction for anti-infective therapy (Wang and Wood, 2016). The second is an “active intervention strategy,” which involves developing therapies to directly “disarm the bomb.” Small-molecule inhibitors targeting key proteins of the bacterial SOS response pathway (such as RecA) are highly promising “anti-virulence” drugs. Such drugs aim to prevent prophage activation, thereby inhibiting the expression and dissemination of virulence genes without exerting bactericidal selection pressure, buying time for the host immune system to clear the pathogen (Shearwin et al., 1998; Li et al., 2023).

### 4.3 Future research directions: from phenomenon to precision intervention

Although the “implosion hypothesis” provides a powerful theoretical framework, substantial work is still needed to translate it into precise clinical applications. First, current technologies struggle to differentiate and quantify lysogenic versus lytic phages within the complex gut environment. Therefore, developing new techniques—combining metagenomics, metatranscriptomics, and single-virus/single-cell sequencing—to precisely monitor prophage activation status is a top research priority. Second, there is a need to establish more complex *in vitro* co-culture models (such as organ-on-a-chip) or humanized mouse models that can better simulate the *in vivo* “implosion” process. These models would allow for the systematic validation of interactions between different stress signals, different prophages, and different host genetic backgrounds. Finally, the downstream effects of phage DNA activating innate immune receptors like TLR9, and their net benefit or detriment in different disease contexts, require more in-depth investigation. Answering these questions will be key to moving the field from descriptive phenomena to mechanistic understanding and precision intervention.

## 5 Conclusion

In summary, prophages are not inert “passengers” in bacterial genomes; they are key dynamic elements activated by enteric infection stress. The “Prophage-Mediated Implosion Hypothesis” proposed here places this process at the center of enteric infection pathophysiology, demonstrating how it transforms the commensal microbiota from a defensive barrier into an endogenous disease amplifier. This framework integrates multidisciplinary evidence, offers a robust explanation for antibiotic-associated complications and infection severity heterogeneity, and points toward next-generation therapeutic strategies that target microbiome stability.
